# Sub-optimality in motor planning is not improved by explicit observation of motor uncertainty

**DOI:** 10.1038/s41598-019-50901-x

**Published:** 2019-10-16

**Authors:** Keiji Ota, Masahiro Shinya, Laurence T. Maloney, Kazutoshi Kudo

**Affiliations:** 1 Department of Psychology, New York University, New York, USA; 2 Center for Neural Science, New York University, New York, USA; 3 Institute of Engineering, Tokyo University of Agriculture and Technology, Tokyo, Japan; 4 Research Fellow of Japan Society for the Promotion of Science, Tokyo, Japan; 5 Department of Human Sciences, Graduate School of Integrated Arts and Sciences, Hiroshima University, Hiroshima, Japan; 6 Laboratory of Sports Sciences, Department of Life Sciences, Graduate School of Arts and Sciences, The University of Tokyo, Tokyo, Japan; 7 Interfaculty Initiative in Information Studies, Graduate School of Interdisciplinary Information Studies, The University of Tokyo, Tokyo, Japan

**Keywords:** Decision, Human behaviour

## Abstract

To make optimal decisions under risk, one must correctly weight potential rewards and penalties by the probabilities of receiving them. In motor decision tasks, the uncertainty in outcome is a consequence of motor uncertainty. When participants perform suboptimally as they often do in such tasks, it could be because they have insufficient information about their motor uncertainty: with more information, their performance could converge to optimal as they learn their own motor uncertainty. Alternatively, their suboptimal performance may reflect an inability to make use of the information they have or even to perform the correct computations. To discriminate between these two possibilities, we performed an experiment spanning two days. On the first day, all participants performed a reaching task with trial-by-trial feedback of motor error. At the end of the day, their aim points were still typically suboptimal. On the second day participants were divided into two groups one of which repeated the task of the first day and the other of which repeated the task but were intermittently given additional information summarizing their motor errors. Participants receiving additional information did not perform significantly better than those who did not.

## Introduction

In games played on a dart board, the player must typically consider not just what he is aiming for but also what he might hit instead as a consequence of his own motor error. Any choice of aim point induces probabilities of hitting in every region of the board or even off the board and – if we assign values to each possible outcome – we can attempt to work out the optimal aim point maximizing expected gain^1,2^. This computation is an application of Bayesian decision theory (BDT), a mathematical framework used to model optimal performance when there is known uncertainty about the outcomes of any actions^3–5^.

For almost two decades, researchers have compared human performance in motor decision tasks with optimal Bayesian decision-theoretic performance. In many studies, human performance in motor planning is consistent with optimal performance maximizing expected reward^1,2,6,7^, but in others, it is not^8–16^. In most studies, participants are allowed to learn their motor uncertainty gradually, trial-by-trial, during a training period and one might hypothesize that any sub-optimal performance observed would vanish with enough practice. But in one task even nine days of practice with trial-by-trial feedback of motor error did not result in performance converging to near optimal^13^ A slightly different explanation is that there are working memory limits on the amount of feedback information that can be retained or used in computing an optimal aim point. In both cases, the participant correctly performs Bayesian computations but on insufficient or limited memory capacity.

Other recent work suggests an alternative explanation: participants are sub-optimal because they do not use the available feedback information correctly to compute optimal aim points. There is, for example, evidence suggesting that humans estimates of the probability density functions of motor error are systematically distorted^17–19^. This mismatch between an actual and internal representation of a motor distribution leads in turn to error in calculating the optimal motor plan^18^: no amount of information will lead to near-optimal performance. Herbert A. Simon first suggested that human economic activity may be suboptimal as a consequence of limited information processing and limited computational facilities^20,21^. If there are such limits, then participants can at best optimize their performance constrained by these limits, a form of “bounded rationality” in his terms. Here we consider the possibility that limits on the capacity of working memory underly observed sub-optimality in motor decision tasks with the alternative being a failure to correctly compute optimal aim points despite having sufficient feedback information to do so.

If participants fail because they do not have enough feedback information or cannot retain enough trial-by-trial feedback information in working memory, then providing additional information in a form not limited by working memory should lead to performance closer to optimal. We report an experiment in which all participants performed a motor decision task with trial-by-trial feedback in two sessions over two days. In one condition, on the second day, we added a second source of information about motor uncertainty (a “Display”), removing the need to remember trial-by-trial feedback by explicitly displaying all the motor outputs which participants performed previously. The Display was shown every 50 trials. We, in effect, replaced their working memory summary of feedback. Previous work demonstrates that participants can make use of such displays in visuo-motor tasks^22–25^.

We also examined performance in a Full Information condition in which participants were given a near-perfect graphical representation of the probability density function of their motor error (see, the Methods section for details). In effect, they were given more information about their motor error than would be available through trial-by-trial feedback alone or trial-by-trial feedback with intermittent displays. We still found that performance was suboptimal under this source of information. These findings indicate that inaccurate representation of motor uncertainty triggered by a lack of feedback possibly due to limited memory capacity is not the primary source of suboptimality in motor planning—a failure to use available information correctly is the more plausible explanation.

## Results

### Experimental design and calculation of risk-sensitivity

The experiment comprised three tasks (Training, Decision, Full Information) in two sessions over two days. We initially focus on the Decision task. Descriptions of the Training task can be found in *Design and procedure*. We describe the Full Information task below.

**Figure 1:**
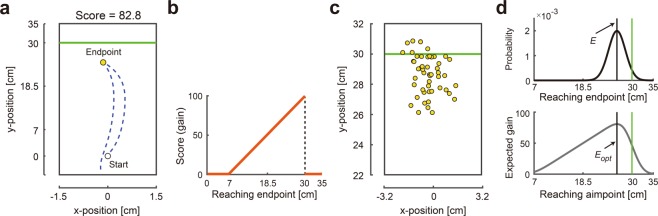
Experimental setup and model assumptions. **(a)** Trajectory of a reaching movement. The participants made a quick out-and-back reaching movement by moving forward from an initial position (white circle) and returning to the initial position. The maximum y-position was recorded as the outcome of reaching movement (i.e., reaching endpoint, yellow circle). **(b)** Asymmetric gain function. The score for each trial was determined by the outcome. A higher score was awarded by reaching further; however, the score fell to 0 points when reaching over the green boundary line (30 cm). **(c)** Display condition. The participants were shown the motor uncertainty comprising their own endpoints after each block (50 trials). **(d)** Model assumptions. The upper panel shows the probability density function of the reaching endpoint for given an aim point (black vertical line). By integrating the probability density function over the asymmetric gain function, the expected gain can be calculated as a function of the aim point (lower panel). The aim point that maximizes the expected gain (black vertical line) *E*_*opt*_ is defined as the optimal and risk-neutral strategy. The upper panel also illustrates the probability density function when the expected gain was maximized.

The motor decision task used in this study was similar to those used in previous studies that showed sub-optimal motor planning^11–13^. In our task, participants made a quick out-and-back reaching movement (moving forward from the start position and returning to the start) on a pen-tablet (Fig. 1a). As the task was speeded, the trajectory was not fully under the control of the participant. Participants manipulated a cursor presented on a vertical display by using a digitized pen on a pen-tablet. They could earn points on each trial and were instructed to maximize the total number of points earned. The participants started their movement after a green boundary line appeared on the display (set 30 cm forward from the start position) and were required to complete it within a time limit. If they failed to complete the movement within the time limit, the trial terminated (“*timeout*”), the participant received a warning message and sound signal. If the participant completed the trial within the time limit, we recorded the point on the trajectory with the maximum y-position (marked as a yellow circle in Fig. 1a). Horizontal displacement played no role. We refer to this maximum y-point as the *outcome* (i.e., endpoint of reaching movement).

The participants received a reward determined by the outcome as specified by the gain function (Fig. 1b). The closer the outcome to the green boundary line, the higher the reward unless the outcome fell above the green line. In that case (a *mistrial*), no reward was received. The participant received the maximum reward (i.e., 100 points) if the outcome fell on the green line to graphical precision.

Due to the inherent noise of the motor system, the outcome of reaching movement could vary every trial even if the participants planned to make movements with the same distance. This motor uncertainty could be approximated by a Gaussian distribution centered at an aim point^1,2^ (Fig. 1d upper panel). Bayesian decision theory predicts the optimal aim point that maximizes the expected gain by combining the motor uncertainty with the asymmetric gain function (Fig. 1d, lower panel; see *Model assumptions*). We compared the optimal aim point *E*_*opt*_ with the participant’s actual aim point *E*_*obs*_ and defined this difference, *E*_*obs*_ − *E*_*opt*_, as their risk-sensitivity. A positive value of risk-sensitivity (*E*_*obs*_ > *E*_*opt*_) indicated that the participants employed a suboptimal *risk-seeking* strategy that led to a higher probability of mistrial but typically larger rewards if a mistrial did not occur. In contrast, a negative value of risk-sensitivity (*E*_*obs*_ < *E*_*opt*_) indicated that the participants employed a suboptimal *risk-averse* strategy since it led to a lower reward but with a low probability of mistrial. A risk-sensitivity of 0 corresponded to the optimal *risk-neutral* strategy. The reaching trajectory was drawn on the screen and the outcome marked as a yellow circle. The participants could estimate their own motor uncertainty by observing this feedback.

All participants first completed 7 blocks of 50 trials of the motor decision task receiving trial-by-trial feedback. A few days later, these participants were randomly assigned to either a Display or No Display condition. The participants in the No-Display condition completed 7 blocks of 50 trials of the motor decision task just as on the first day. Participants in the Display condition also completed 7 blocks of 50 trials of the motor decision task with trial-by-trial feedback but, after each block of 50 trials, they received a graphical summary of their outcomes in the preceding 50 trials (Fig. 1c). This feedback made the motor uncertainty explicitly available to the participants (see *Design and procedure*).

### Effect of observing motor uncertainty on motor planning

**Figure 2:**
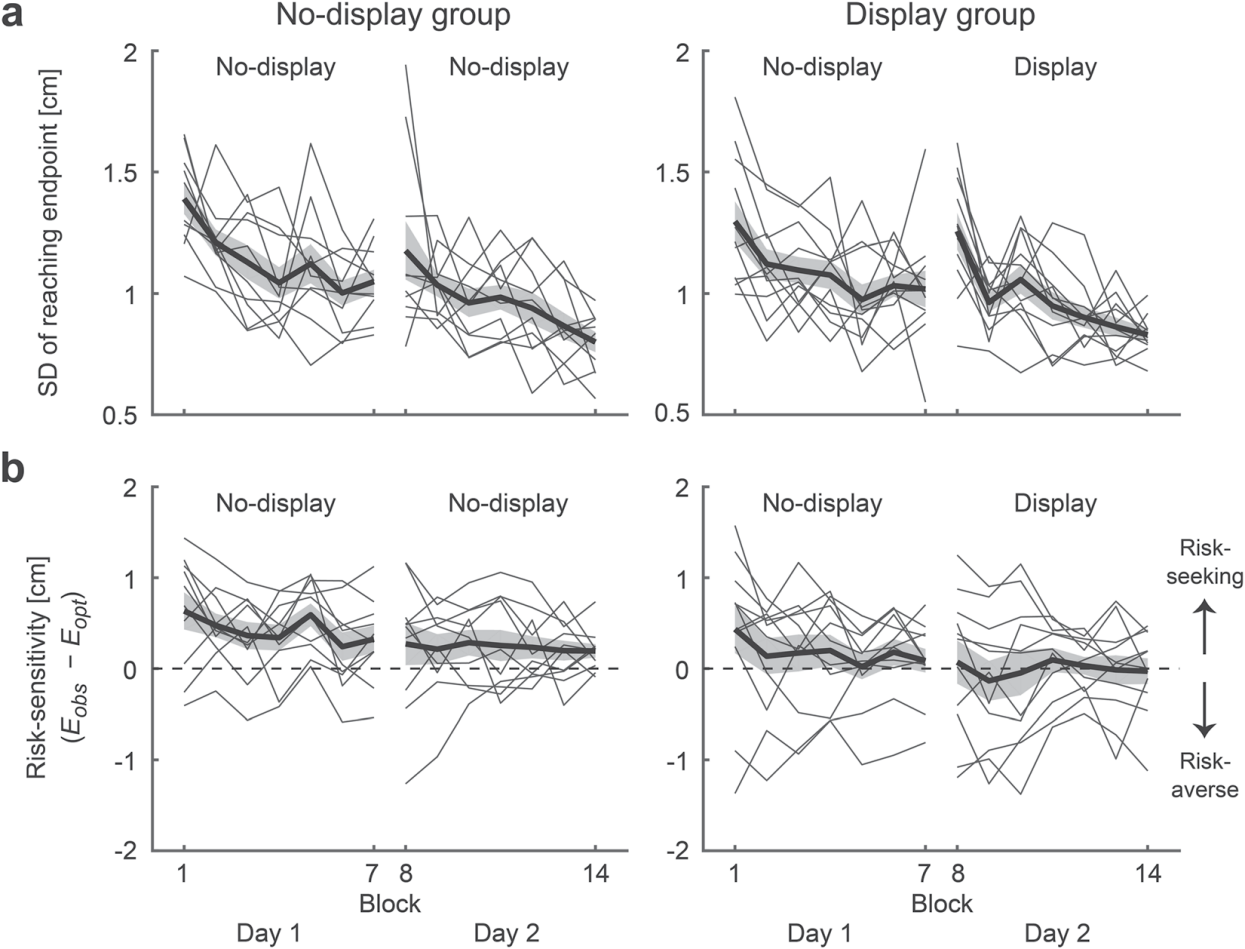
Effect of feedback of the motor uncertainty on performance indexes. Time series of the standard deviation (SD) of the reaching endpoints **(a)** and the risk-sensitivity **(b)** for the no-display and display groups. The thick black line represents the average across the participants, and the shaded area represents the standard error of the mean. Thin lines indicate data from each participant. The SD of reaching the endpoint decreased with practice, indicating that the reaching accuracy was improved. In both groups, each participant’s risk-sensitivity value (thin line) did not converge to 0 (i.e., risk-neutral) on day 2. Therefore, the feedback of motor uncertainty had a small effect on changing the participant’s risk-sensitivity in motor decision-making.

We first investigated the difference in reaching variability between the display and no-display groups. Figure 2a illustrates a time series of the standard deviation (SD) of the reaching endpoint. A two-way (2 [no-display and display groups] * 14 [blocks]) mixed-effects ANOVA showed a significant main effect of a block (*F* [5.86, 111.34] = 15.11, *p* = 0.001, *η*^2^ = 0.33) but no main effect of a group (*F* [1, 19] = 0.14, *p* = 0.71, *η*^2^ = 0.00) or interaction (*F* [5.86, 111.34] = 0.93, *p* = 0.47, *η*^2^ = 0.02). The results suggested a learning effect on movement variability but no difference between the groups.

We then focused on the signed risk-sensitivity value (Fig. 2b). In the no-display group, a risk-sensitivity value for each participant (thin lines in Fig. 2b, left panel) did not converge to a risk-neutral value (i.e., 0) from day 1 (no-display condition) to day 2 (no-display condition). This finding suggested that, in line with a previous study^13^, the suboptimal risk-seeking or risk-averse strategy remained consistent when the participants practiced the task without seeing their own motor uncertainty. If memorizing outcomes of reaching movement is the principal source of sub-optimality in performance, the risk-sensitivity value for each participant would converge to 0 when the motor uncertainty was explicitly available. However, we did not find such a trend in the display group (thin lines in Fig. 2b, right panel). We also did not find evidence that the display condition made the participants more risk-averse or risk-seeking since a two-way (2 [no-display and display groups] × 6 [blocks 9–14]) mixed-effects ANOVA on the signed risk-sensitivity showed neither a main effect of a group (*F* [1, 19] = 1.71, *p* = 0.21, *η*^2^ = 0.06), main effect of a block (*F* [5, 95] = 0.52, *p* = 0.76, *η*^2^ = 0.01), nor a significant interaction (*F* [5, 95] = 0.35, *p* = 0.88, *η*^2^ = 0.00).

**Figure 3:**
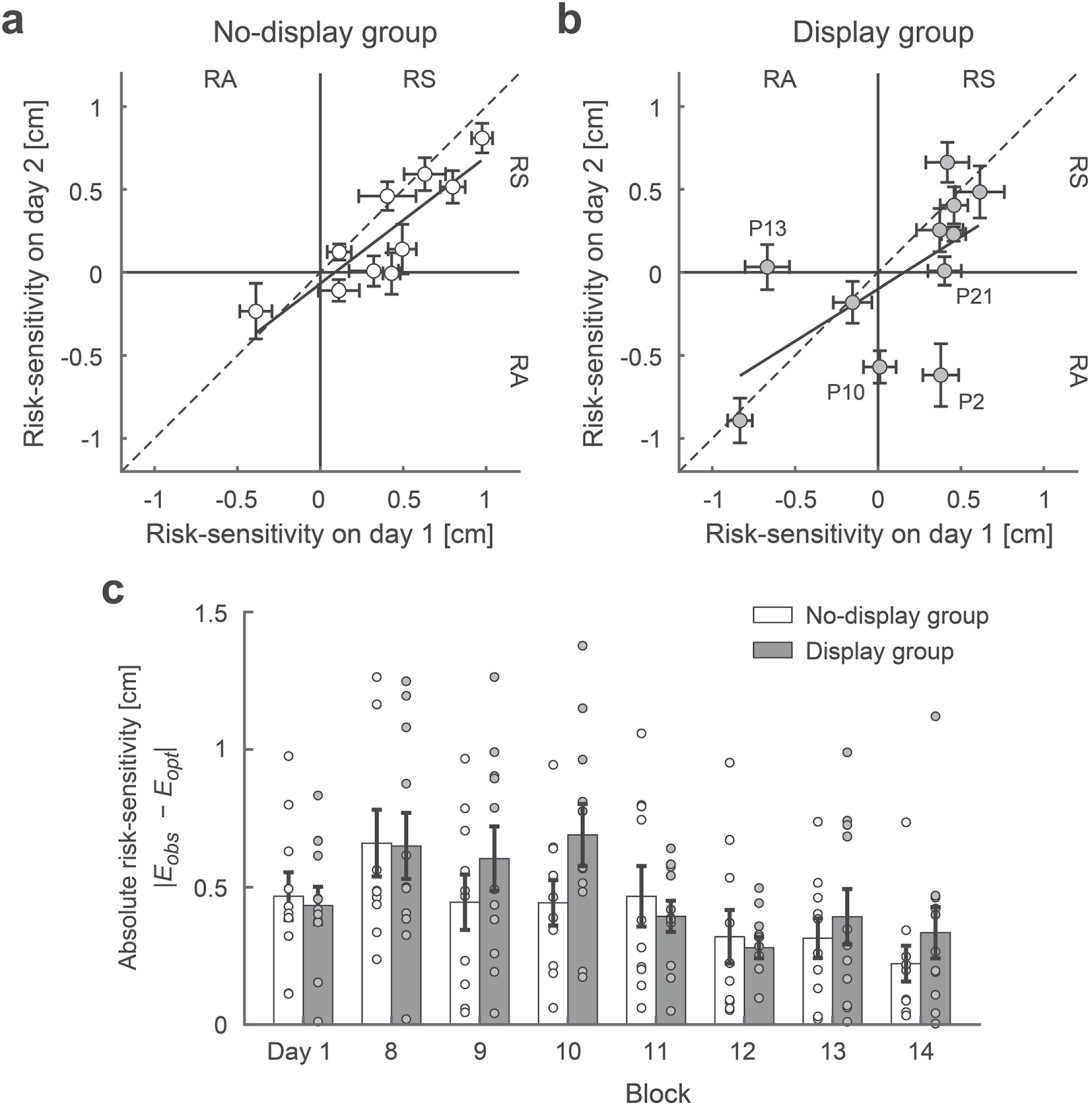
Suboptimal behaviour was retained after the feedback of the motor uncertainty. The average risk-sensitivity on day 1 (average of blocks 2–7) is plotted against that on day 2 (blocks 9–14) for the no-display group **(a)** and display group **(b)**. Horizontal and vertical error bars represent the standard errors of the mean within day 1 and day 2, respectively. Data points that fall in the top right quadrant correspond to the risk-seeking to risk-seeking strategy, whereas data points that fall in the bottom left quadrant correspond to the risk-averse to risk-averse strategy. In the no-display group, a slope of 0.76 in a linear regression line suggests a consistent tendency of adopting the risk-seeking or risk-averse strategy. In the display group, although a few participants (P2, P10, P13, and P21) changed their strategy, the slope of the regression line was still significantly positive (slope = 0.63). **(c)** The absolute risk-sensitivity, as an index of the degree of suboptimality, is plotted for day 1 (averaged over blocks 2–7) and each block of day 2. Error bars represent the standard errors of the mean. Circles represent data from each participant. There was no significant difference between the no-display and display groups, suggesting no significant effect of explicitly presenting motor uncertainty on a suboptimal motor plan.

Figure 3a,b illustrates the average risk-sensitivity on day 1 (blocks 2–7) against that on day 2 (blocks 9–14). In the no-display group, a least-squares linear regression between day 1 and day 2 resulted in a slope of 0.76 (95% CI = [0.40, 1.13], *R*^2^ = 0.74, *p < *0.001, Fig. 3a), which suggested that risk-seeking or risk-averse strategies persisted from day 1 to day 2. Additionally, in the display group, we found a significant positive slope of the regression line (slope = 0.63, 95% CI = [0.02, 1.24], *R*^2^ = 0.38, *p = *0.044, Fig. 3b). There was no significant difference in the slope between groups (analysis of covariance: *F* [1, 17] = 0.15, *p* = 0.71), consistent with the null hypothesis.

In Fig. 3c, we further plotted the absolute risk-sensitivity values ($|{E}_{obs}-{E}_{opt}|$) on day 1 (averaged over blocks 2–7) and for each block on day 2 to determine the degree of suboptimality in motor planning. As a baseline performance, there was no significant difference between the absolute risk-sensitivity in the display and no-display groups (independent *t*-test: *t* [19] = 0.31, *p* = 0.76). On day 2, a two-way (2 [no-display and display groups] × 6 [blocks 9–14]) mixed-effects ANOVA showed neither a main effect of a group (*F* [1, 19] = 0.70, *p* = 0.42, *η*^2^ = 0.02) nor a significant interaction (*F* [5, 95] = 1.70, *p* = 0.14, *η*^2^ = 0.03). Furthermore, a one-sample *t*-test revealed that there was a significant difference the absolute risk-sensitivity value on the 14th block and a risk-neutral value (i.e., 0) both in the display (*t* [10] = 3.59, *p = *0.005, *d = *1.60) and no-display groups (*t* [9] = 3.40, *p = *0.008, *d = *1.60).

We addressed the possibility that the participants in the display group rapidly forgot the observed motor uncertainty after each display. If so, we might expect to see a transient movement toward risk-neutral (optimal) after each display presentation. Accordingly, we compared the participant’s performance in the last 10 trials of each block (pre-display), with that in the first 10 trials of the following block (post-display). We did not find any improvements in the absolute risk-sensitivity immediately after the display (Suppl. Fig. 1). Therefore, forgetting the information conveyed by the display was not a reason why the feedback did not have a significant effect on motor decisions.

We are failing to reject a null hypothesis and so we need a more sensitive way of measuring the support for and against the null hypothesis. To do so, we calculated Bayes factors which are an index of the relative strength of evidence for the hypothesis of a feedback effect to the null hypothesis of no feedback effect^26^. A Bayes factors of above 3 indicates substantial evidence for the alternative hypothesis (H1: the difference between groups) over the null hypothesis (H0: no difference between groups) and below 0.33 indicates substantial evidence for the null hypothesis (H0) over the alternative hypothesis (H1)^27^. A value in the intermediate range (0.33 and 3) supports neither hypothesis.

In computing Bayes factors, we modeled the predictions of H1 as a random variable drawn from a uniform distribution with a lower limit and an upper limit bracketing the range of effect size that could be expected^26^. The average of absolute risk-sensitivity in the last 6 blocks (block 9–14) across the participants of the no-display group was 0.30 cm. If the feedback of motor uncertainty completely improved the suboptimality, we would expect the absolute risk-sensitivity in the display group to be 0 (i.e., optimal). If, on the other hand, the feedback was not effective at all, the absolute risk-sensitivity in the display group would be the same value as that in the no-display group. Thus, the predictions of population difference between group means ranged from 0 (=0.30 − 0.30 in case of no effect) to 0.30 (=0.30 − 0 [optimal value] in case of the maximum effect).

We found that the average of absolute risk-sensitivity in the last 6 blocks of the display group was 0.39 cm and that the mean difference between groups was −0.09 (non-significantly different between groups: *t* [19] = −0.78, *p* = 0.44, standard error = 0.12). The Bayes factor analysis (mean = −0.09, SE = 0.12, uniform from 0 to 0.30, http://www.lifesci.sussex.ac.uk/home/Zoltan_Dienes/inference/Bayes.htm) provided a Bayes factor of 0.30, indicating the substantial evidence in favor of the null hypothesis (i.e., the data was 3 times more likely to reflect the null than the alternative hypothesis). Taken together, these findings suggested that displaying the motor uncertainty had little or no effect on a participant’s suboptimal motor planning.

### The full information task

**Figure 4:**
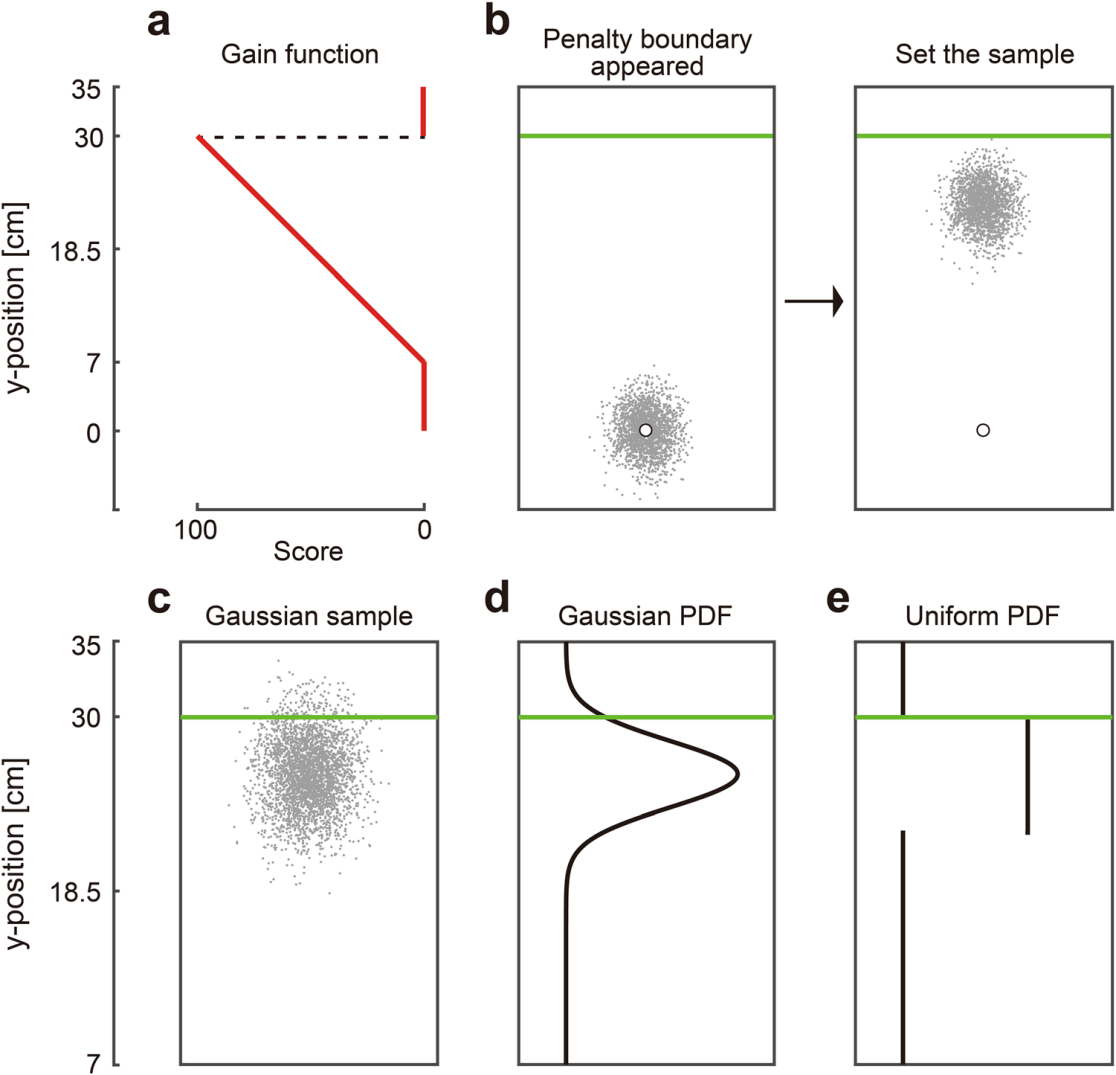
Full information task. **(a)** Gain function. (**b)** Trial sequence. The participants first moved the sample or probability density function (PDF) to an initial position (blue circle). After the green penalty boundary appeared, they moved the sample or PDF to the position where they thought they could maximize the expected gain for a given sample or PDF. The participants were shown either a sample of a Gaussian distribution **(c)**, a PDF of a Gaussian distribution **(d)**, or a PDF of a uniform distribution **(e)**. The examples in (c), (d), and (e) are set at the theoretical optimal aim point in each distribution. We compared this optimal aim point with the participants’ set point. For example, the example in (b) is set farther from the penalty boundary than the optimal example in (c), showing risk-averse behaviour. The participants completed trials with narrow and wide widths for each distribution.

The results in the decision task suggested that observed sub-optimality is not the result of limitations on short-term memory capacity. Failure to correctly combine reward and probabilities derived from motor uncertainty may be more critical. To explore this point further, in the last part of the experiment (the Full Information Task), we presented graphical representations of the probability density function of motor error and asked the participants to indicate the optimal aim point of those distributions (Fig. 4).

In this full information task, we tested three graphical representations (a large sample from a Gaussian distribution (Fig. 4c), probability density function of the Gaussian distribution (Fig. 4d), and we also presented the probability density function of a uniform distribution as a foil (Fig. 4e)). We included the uniform because the task is remarkably easy with a uniform probability density function (see Suppl. Fig. 2). We presented the graphical displays with two scalings in the vertical direction (“narrow width” and “wide width”). The participants could manipulate the visible sample or PDF by moving the digitized pen which translated the mean of the distribution.

In each trial, the participants moved the mean of the distribution to the start position (Fig. 4b). After the green boundary line (set at 30 cm forward from the start position) was presented, the participants were instructed to move the sample or PDF to the location where they thought they could maximize the expected reward under the asymmetric gain function (Fig. 4a,b). We recorded the setpoint of each trial as a mean of the distribution and defined this variable as a *subjective optimal aim point*. We also calculated a *theoretical optimal aim point* by finding the point that maximizes the expected reward under the asymmetric gain function for a given distribution (see *Model assumptions*).

We first plotted a signed difference between the subjective and theoretical optimal aim points and found that the participants were more risk-averse with the dots drawn from the wide Gaussian distribution than with the PDF of the wide uniform distribution. They were also more risk-averse with the PDF of the wide Gaussian distribution than with the PDF of the wide uniform distribution (Suppl. Fig. 3). To determine how close the participants set the distribution from the optimal point, we calculated the absolute difference between the subjective and theoretical optimal aim points. We ignore whether the difference is positive (risk-seeking) or negative (risk-averse) in the analysis below.

**Figure 5:**
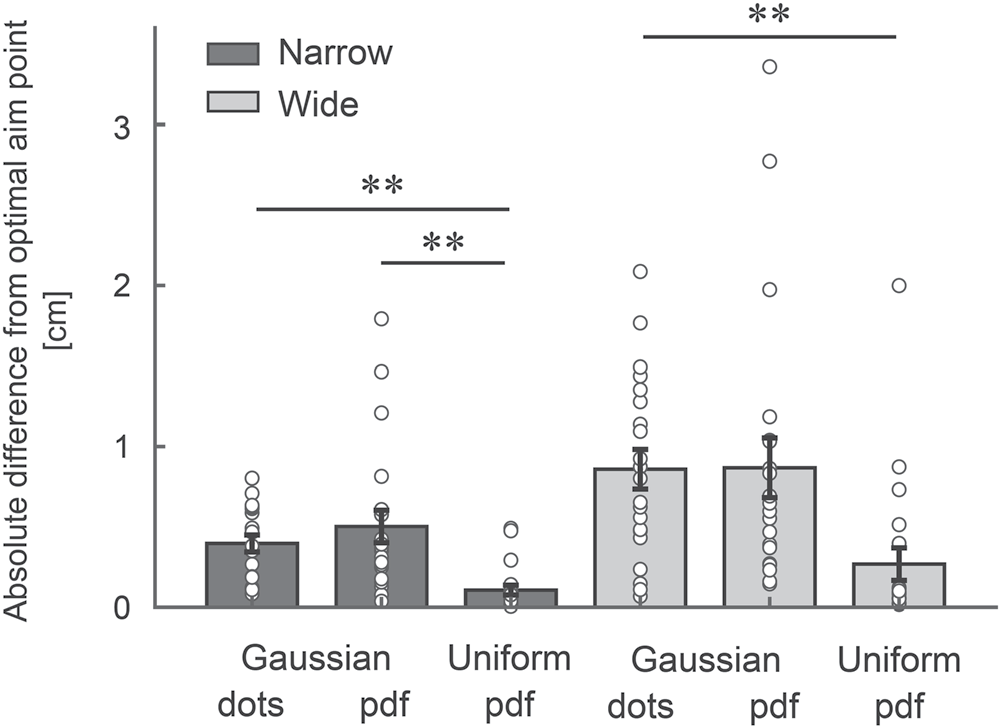
The results of the full information task. The absolute differences between the subjective and theoretical optimal aim points are plotted for each distribution. Error bars represent the standard errors of the mean. Circles denote the data from each participant. There was a smaller deviation from the theoretical solution in the probability density function (PDF) of a uniform distribution than a sample and PDF of Gaussian distribution. The participants showed difficulty in computing a Bayes optimal solution when manipulating a Gaussian distribution. **Represents *p* < 0.01.

We pooled the data between the display and no-display groups because a three-way (2 [groups] × 3 [sample of Gaussian, PDF of Gaussian, and PDF of uniform distribution] × 2 [narrow and wide]) mixed-effects ANOVA did not show a main effect of the group (*F* [1, 19] = 1.45, *p* = 0.24, *η*^2^ = 0.02). Figure 5 illustrates the absolute difference of the six distributions (averaged over all the participants). A two-way (3 [distribution types] × 2 [width of distribution]) within-subject ANOVA yielded a main effect of width of distribution (*F* [1, 20] = 21.28, *p* = 0.001, *η*^2^ = 0.11), suggesting that the participants showed a larger deviation from the theoretical optimal aim point in the wide condition (Fig. 5). ANOVA also showed a main effect of distribution type (*F* [1.57, 31.30] = 8.51, *p* = 0.002, *η*^2^ = 0.19). We found a significantly smaller deviation between the PDF of a uniform distribution and the sample or PDF of a Gaussian distribution in the narrow condition (*ps* < 0.006, Bonferroni correction) and between the PDF of a uniform distribution and the sample of a Gaussian distribution in the wide condition (*p* = 0.008, Bonferroni correction). Therefore, the participants’ performance was more sub-optimal when combining the reward information with a Gaussian distribution than with a uniform distribution. This inability to correctly combine reward and probabilities might be one of the main constraints in optimal motor decision-making.

## Discussion

Previous studies have shown that humans have difficulty in making optimal and risk-neutral motor decisions on the basis of their own motor uncertainty^8–16^. Here, we investigated whether the suboptimality in motor planning could be improved by explicitly providing the motor uncertainty and removing the need to remember the outcomes of motor planning. We found that the participants still performed suboptimally and that the degree of suboptimality was similar between the groups with or without the feedback of the motor uncertainty (Fig. 3c).

Importantly, a target (an aim point) was not explicitly given to the participants in the decision or full information tasks. The optimal aim point maximizing expected gain had to be learned and was determined by the participant’s own motor uncertainty. Knowledge of the motor uncertainty could be accumulated by observing the result of the outcome of motor planning (i.e., reaching endpoint) in each trial. Thus, the participants were required to update their aim point based on constructed knowledge of the motor uncertainty in a block-by-block manner. A previous study showed that such learning of an aim point was not established after practice for 45 blocks (2250 trials)^13^. It would be difficult to remember all the outcomes and learn statistical properties of motor uncertainty because the capacity of short-term memory is limited^28^. In fact, the internal representation of one’s own motor error deviates from an actual (Gaussian) distribution^17–19,29^. We thus allowed the participants to see their own motor uncertainty. Although the motor uncertainty was available to the participants, 7 of 11 participants retained the risk-seeking or risk-averse strategy (Fig. 3b). Only a few participants switched their strategy after the presentation (P2, P10, P13, and P21 in Fig. 3b). Therefore, even if the participants were given their own motor uncertainty in symbolic form, their decisions deviated markedly from optimal.

Why did additional information in the display condition not benefit motor planning? Perhaps participants become “habituated” to single-endpoint feedback and do not bother to use the additional information available in the displays on Day 2? The key question is why does the motor planning system fail to use information from the display and combine it with trial-to-trial information when both are available. One possibility is that they are over-confident concerning their estimates of motor error and choose to make no use of further information. Another possibility is that the motor system simply cannot combine trial-by-trial information with the kind of display we employed. That is, while information from either source alone is beneficial: information from both sources cannot be combined. Further studies to test these claims and similar claims are needed.

In the full information task, we further asked the participants to indicate the optimal aim point for a given graphical representation of uniform or Gaussian distribution (Fig. 4). In a Gaussian distribution, the probability of obtaining a higher reward increased as the participants set the mean of the distribution closer to the penalty boundary. At the same time, the risk of the penalty also increased (Suppl. Fig. 4). Therefore, when integrating a Gaussian distribution over the asymmetric gain function, the participants needed to consider the trade-off between the chance of higher reward and risk of penalty. The optimal penalty ratio was 3.9% in the case of *σ* = 2 cm (as shown in Fig. 4c,d). In contrast, there was no such trade-off when manipulating a uniform distribution: the probability of obtaining higher reward first increased when the participants set the mean closer to the boundary, but it did not increase any more after the edge of the uniform distribution came to the boundary line (for details of this simulation, see Suppl. Fig. 4). The optimal strategy is more intuitive because it requires one to adjust the edge of the uniform distribution on the penalty boundary and to set the penalty ratio to 0% (as shown in Fig. 4e). The participants were better at finding this theoretically optimal aim point in a uniform distribution than in a Gaussian distribution (Fig. 5). These findings suggest that the ability to perform a Bayes computation while considering a trade-off between reward and risk was limited and might be one of the primary constraints to learning the aim point.

Another possible explanation of suboptimality is a motor cost (or motor energy). The original application of Bayesian decision theory to motor tasks included an explicit term for the “biological cost” of an action^1,2^. If two movement plans offered the same reward, the movement plan that used the least energy would be chosen. Most applications of the theory involve experiments where biological cost is roughly constant across possible movement plans and does not affect the selection of the optimal movement plan^1,2,7,11–13,15,16^. The task in this study is of this type with the 550 ms timeout intended to control the energy cost. In fact, the majority of our participants even sought a higher one-trial gain by reaching further distance than the optimal strategy (Fig. 3a,b). Therefore, a motor cost is not a likely explanation of observed suboptimality in our task.

Similar (but not the same) tasks have been used in the studies of Bayesian inference. The goal of those tasks is to estimate a quantity of unknown stimuli, such as speed^30^, position^24,31–33^, orientation^34–36^, interval timing^37–39^, temporal order^40,41^, and convex-concave shape^42^. To make such estimations, participants are required to combine prior knowledge of a stimulus quantity and current sensory information and read out the highest posterior probability (which is called *maximum a posteriori* estimation). Whether humans can perform such a Bayes’ optimal inference depends on the complexity of the prior distribution. If a prior distribution is a simple Gaussian distribution, humans have been shown to perform optimally^24,32,33,38–40,43^. It requires a few hundred trials to learn the mean and variance in a Gaussian (prior) distribution^39,43^. A more complex distribution presents a greater challenge for human probabilistic inference. When the prior distribution becomes bimodal, training requires thousands of trials^32^ or performance remains suboptimal after a few hundred trials^37^. Acerbi *et al*.^31^ used a unique paradigm in which the complex prior was explicitly provided to participants. The results suggested that the suboptimality in probabilistic inference was due to a problem of learning statistical features of the complex prior rather than computing the complex probabilistic information. In contrast to those tasks in which a stimulus (or prior distribution) is externally provided by experimenters, the prior distribution is internally determined by one’s own motor system in motor tasks. Human’s internal representations have been shown to be inaccurate even with a simple Gaussian distribution^17–19^. Humans also have an optimistic prediction of success for their own actions^44^. Therefore, there was a possibility that the suboptimality in motor planning was solved by presenting the motor distribution but the suboptimality was still retained (Fig. 3). The factors of suboptimality in motor decision tasks may be different from those in probability inference tasks.

The obtained results have important implications in the sports field. Visualizing of motor performance has become popular and available due to recent technical developments. For example, players can see their own (or an opponent’s) landing positions of service/strokes in tennis (Hawk-Eye system^45,46^) and the distribution of pitches in a baseball (PITCHf/x^47,48^). However, our results suggest that solely presenting those distributions might be insufficient. Together with a presentation of the performance distribution, it would be important for coaches and advisers to evaluate whether a tendency of the distribution is biased towards a risk-seeking or risk-averse strategy to optimize a player’s aiming performance.

## Methods

### Participants

This study was approved by the Ethics Committee of the Graduate School of Arts and Sciences at the University of Tokyo, and was carried out in accordance with their guidance. Subjects provided written informed consent and were unaware of the purpose of the experiment. We recruited 21 healthy right-handed adults for the experiment. Eleven (9 males; 20.5 ± 1.6 years) and ten participants (8 males; 21.7 ± 2.4 years) were randomly assigned to the display and no-display groups, respectively. There were 15 previous studies of motor decision-making similar to ours; eight included 3–10 participants^1,2,6–8,15,24,32^ per condition and seven included more, up to 20^9–14,16^. We thus determined our sample size of 10 participants in one group.

### Apparatus

The participants performed a quick out-and-back reaching movement holding a digitized pen on a pen-tablet (Wacom, Intuos 4 Extra Large; workspace: 488 × 305 mm). They used their dominant (right) hand to perform the reaching movement. The position of the digitized pen was sampled at ~144 Hz with a spatial resolution of 0.01 mm. The pen position was also transformed to a cursor position presented on a vertical screen (Asus, VG-248QE; size: 24 inches, refresh rate: ~144 Hz) with a maximum delay of 6.9 ms. The participants were instructed to manipulate the cursor by moving the pen; their hand and the pen were covered with a box over the pen-tablet. The distance to the screen was 75 cm, and the scale of the pen and cursor position was 1:1. All stimuli were controlled using the Psychophysics Toolbox^49,50^.

### Experimental task

There were three tasks: a training task, a decision task, and a full information task. To begin each trial in the training and decision tasks, the participants moved a blue cursor (radius: 0.3 cm) to an initial white position (radius: 0.4 cm) presented on the vertical screen (Fig. 1a). After a 1-s delay, a horizontal white line (width: 0.1 cm) appeared forward from the initial position (see below for details of distance). After random intervals of 0.8–1.2 s, the white line turned green, which indicated the “go” signal and “boundary line”. The participants then made a quick out-and-back reaching movement with which the cursor was rapidly moved forward and returned below the initial position (Fig. 1a). The cursor was visible during the movement. We recorded the endpoint of each movement as the maximum y-position (Fig. 1a). If the participants did not return the cursor within the time-out interval, the message “Time out. More quickly!” appeared with a warning tone. If the participants successfully returned the cursor, a yellow cursor (radius: 0.3 cm) appeared at the maximum y-position for 2 s. We refer to this yellow cursor as the *outcome* of the trial. After the feedback period, the participants proceeded to the next trial. The training and decision tasks differed in the rewards associated with the outcome as explained next.

### Training task

In the training task, the participants were in effect asked to place the outcome as close as possible to the green boundary line. After each movement, if the outcome (yellow cursor) overlapped with the green boundary line, the message “Hit!” appeared with a pleasant sound. The line was set at three distances (11, 23, and 35 cm from the initial position). The time-out intervals imposed were 400, 500, and 600 ms, respectively. The trials in which the participant timed out were repeated.

### Decision task

In the decision task, the green boundary line was set at 30 cm from the initial position. The time-out interval was 550 ms. The participants scored points depending on the location of the outcome with respect to the green line on trials where a mistrial did not occur. When a mistrial occurred, a “Miss!” message was presented with a flashing red lamp and an unpleasant alarm. Zero points would have been awarded if the outcome was within 7 cm of the initial position, but no such instances occurred. Otherwise, if the outcome fell above the green line, the participant received no reward. If it fell on or below the green line, then the closer the outcome was to the green boundary line, the more points were provided. See Fig. 1b for a specification of the relationship between the location of the outcome and reward. Using an asymmetric gain function is valid because this function can reveal suboptimal risk-seeking or risk-neutral behaviour^11^. If we used a function whose gain was distributed symmetrically around the boundary line, we could not measure a subjects’ risk-sensitivity because aiming at the line was obviously optimal for all subjects.

Participants received feedback. In the feedback period, the participants were shown a score for that trial, the total score, and a yellow circle at the location of the outcome. The participants were instructed to maximize the total scores in each experimental block (50 trials). They were also told that the total score would be translated into bonus payments (1 Japanese Yen / 100 points) and that exceeding the time-out interval would be penalized (−5 Japanese Yen/time).

There were display and no-display conditions in the decision task. In the display condition, the participants were given feedback for the motor uncertainty comprising the reaching endpoints for 50 trials after each experimental block (Fig. 1c). This feedback lasted for 15 s. The participants were told that the feedback summarized their own endpoints that they had performed. This information removed the need to memorize all the outcomes and made the motor uncertainty explicitly available to the participants. In the no-display condition, the participants were shown a message stating “Please wait” (without showing the motor uncertainty) which lasted for 15 s.

### Full information task

In the full information task, we presented a stimulus intended to represent a probability density function (PDF) and tested the participant’s ability to set it to the optimal aim point. The stimulus could be a bivariate sample from a Gaussian (Fig. 4c), a 1D Gaussian PDF (Fig. 4d), or a 1D Uniform PDF (Fig. 4e). This full information task served as a complement to the decision task, testing participants’ performance with near-perfect representations of motor uncertainty.

As in the decision task, the participants first moved the mean of the distribution to a white start position (Fig. 4b). After a random interval of 0.8–1.2 s, the white line appeared 30 cm above the start position and turned green. After the colour changed, we asked the participants to move the sample or PDF to the location where they thought they could maximize the expected reward under the asymmetric gain function (Fig. 4a,b). There was no time constraint.

We performed two width (narrow and wide) conditions for each distribution. Let *σ*_*V*_ as the standard deviation in the vertical direction of sample/PDF. We defined the *σ*_*V*_ in the narrow condition as the standard deviation of the participant’s reaching endpoint in the last 3 blocks of the decision task (blocks 12–14). Therefore, the sample/PDF in the narrow condition reflected the participant’s own motor uncertainty. The magnitude of the standard deviation was doubled in the wide condition.

In the sample from a Gaussian distribution (Fig. 4c), the participants were shown a sample of dots drawn from an anisotropic bivariate Gaussian distribution with mean $$(\begin{array}{c}0\\ 0\end{array})$$ and covariance $$(\begin{array}{ll}{{\rm{\sigma }}}_{V}^{2}/2 & 0\\ 0 & {\sigma }_{V}^{2}\end{array})$$. The number of dots for a participant ranged from 930 to 1635 and was proportional to *σ*_*V*_. The sample from the Gaussian probability density function gives only an estimate of the true underlying probability density function but the sample size is large enough that we can ignore the distinction. In the PDF with the Gaussian distribution (Fig. 4d), the participants were shown a probability density function drawn from a univariate Gaussian distribution with vertical mean (0) and vertical standard deviation *σ*_*V*_. In the PDF with a uniform distribution (Fig. 4e), a probability density function was drawn from a univariate uniform distribution with vertical mean (*a* + *b*)/2 and vertical standard deviation ${\sigma }_{V}=(b-a)/2\sqrt{3}$.

### Design and procedure

Two (display and no-display) groups of participants came to a laboratory on 2 successive days (the interval between the two days was 1.8 ± 0.9 days). On the first day, two experimental sessions were performed. All participants first performed the training task for 50 trials at each distance (11, 23, and 35 cm) and then performed the no-display condition in the decision task for 7 blocks of 50 trials. Each block was followed by a 2–3 min rest period to reduce fatigue. On the second day, we ran three experimental sessions. First, the no-display group performed the no-display condition, whereas the display group performed the display condition. There were 7 blocks of 50 trials for both groups. Second, all the participants conducted the training task for 30 trials at each distance to ascertain their current reaching variability. Finally, the participants completed the full information task with for 1 block of 8 trials for each of the 6 (3 × 2) sample/PDFs. The order of the sample/PDFs was counterbalanced across the participants. We analyzed the data for the last 6 of the 8 trials.

### Model assumptions

To quantify the participant’s risk-sensitivity, we modelled the optimal mean endpoint (aim point) gain based on Bayesian decision theory^1–4^. We calculated the expected gain *EG*(*E*) for a selected mean endpoint (aim point) by integrating the gain function *G*(*e*) (Fig. 1b) with the probability density function of the reaching endpoint *P*(*e*|*E*).

1 $$EG(E)={\int }_{-\infty }^{\infty }G(e)\cdot P(e|E)de$$

The expected gain as a function of an aim point *E* is illustrated in Fig. 1d (lower panel). The actual reaching endpoint *e* varies in each trial due to sensory-motor noise. We assumed this probability density function (motor uncertainty) as a Gaussian distribution with mean *E* (selected aim point) and variance *σ*^2^ (sensory-motor noise), as shown in Fig. 1d (upper panel).

2 $$P(e|E)=\frac{1}{\sqrt{2\pi {\sigma }^{2}}}\exp [-\,\frac{{(e-E)}^{2}}{2{\sigma }^{2}}]$$

After we obtained a participant’s variability in the reaching endpoint *σ*^2^ in each block, we estimated the optimal mean endpoint *E*_*opt*_ by maximizing the expected gain (Eq. 1). Importantly, the optimal strategy allowed marginal mistrials (3.9% when *σ* = 2 cm; this can be seen as a probability density beyond the green line in the upper panel in Fig. 1d). We compared *E*_*opt*_ with the observed mean endpoint (actual aim point) *E*_*obs*_. If *E*_*obs*_ was closer to the boundary line than *E*_*opt*_, it indicated that the participants employed a suboptimal risk-seeking strategy. If *E*_*obs*_ was further from the boundary line than *E*_*opt*_, the participants employed a suboptimal risk-averse strategy. If *E*_*obs*_ corresponded with *E*_*opt*_, the participants followed an optimal risk-neutral strategy.

We simplified our model based on the assumption that the reaching variability remained constant regardless of the magnitude of the aim point. This assumption was valid because we confirmed that the prediction of the optimal aim point did not differ between the model assuming constant variance and the model assuming proportional variance to the aim point^13^. For a detailed description of our model assumption, see Ota *et al*.^12,13^.

## Supplementary information

Supplementary information

## Data Availability

The data that support the findings of this study are available in Github at https://github.com/keijiota/Explicit-observation-of-motor-uncertainty.

## Code Availability

The code used to generate the data is available in Github at https://github.com/keijiota/Explicit-observation-of-motor-uncertainty.
