# Supplementary material for: Sub-optimality in motor planning is not improved by explicit observation of motor uncertainty
Source: Sci Rep. 2019 Oct 16;9:14850. doi: 10.1038/s41598-019-50901-x (PMC6795881; doi:10.1038/s41598-019-50901-x)
Supplement: Supplementary file 1 — Supplementary information [file 41598_2019_50901_MOESM1_ESM.docx]

## Supplementary information

**Sub-optimality in motor planning is not improved by explicit observation of motor uncertainty**

Keiji Ota*, Masahiro Shinya, Laurence T. Maloney, Kazutoshi Kudo*

*** Correspondence:**Keiji Ota, Email: [keiji.ota@nyu.edu](mailto:keiji.ota@nyu.edu)

Kazutoshi Kudo, Email: [kudo@idaten.c.u-tokyo.ac.jp](mailto:kudo@idaten.c.u-tokyo.ac.jp)

**This PDF file includes:**

Supplementary text

Supplementary Figs. 1 to 4


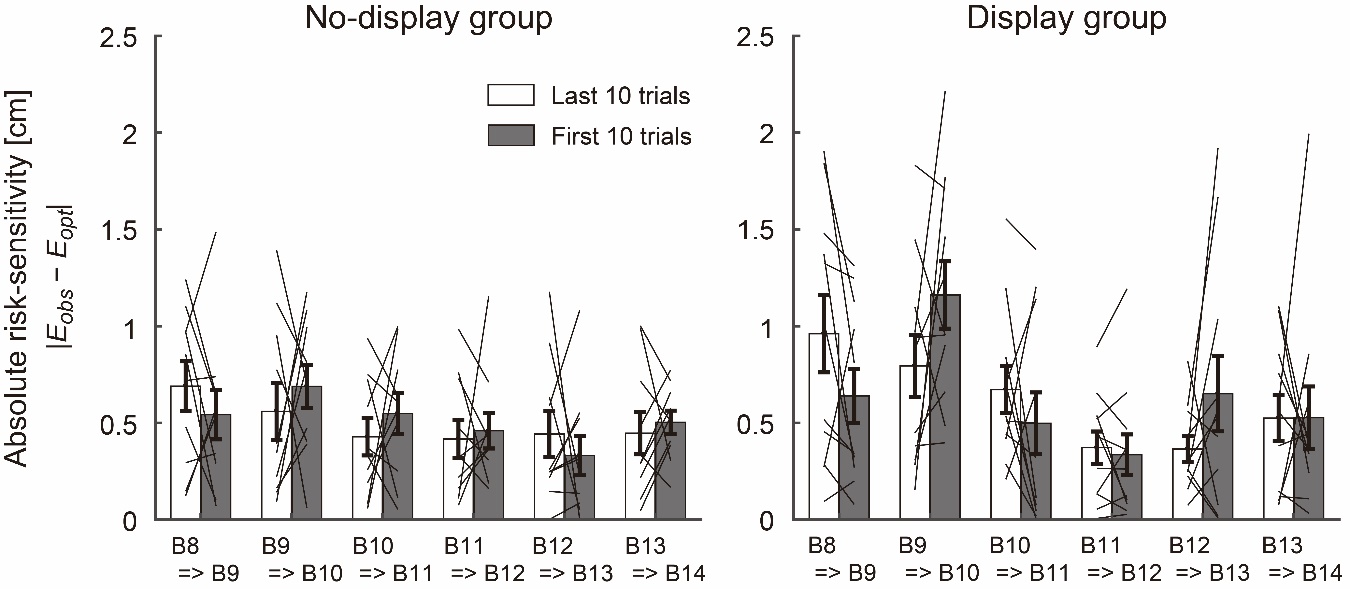


### Supplementary Figure 1. Comparison of performance before and after display.

We plotted the absolute risk-sensitivity in the last 10 trials of each block (pre-display) and that in the first 10 trials of the following block (post-display). Thin lines represent individual data. Bars and error bars represent the mean absolute risk-sensitivity and the standard errors of the mean, respectively. The risk-sensitivity was calculated based on the mean endpoint and the standard deviation of the endpoint in each 10 trial. A three-way (2 [groups] $\times$ 2 [time period: last 10 trials and first 10 trials] $\times$ 6 [blocks from 8 to 9 – from 13 to 14]) mixed-effects ANOVA showed neither a main effect of first and last 10 trials (*F* [1, 19] = 0.08, *p* = 0.78, $\eta^{2}$ = 0.00) nor a significant interaction of the time period and the group (*F* [1, 19] = 0.00, *p* = 0.97, $\eta^{2}$ = 0.00). The results suggest no significant improvement in the absolute risk-sensitivity immediately after the display.


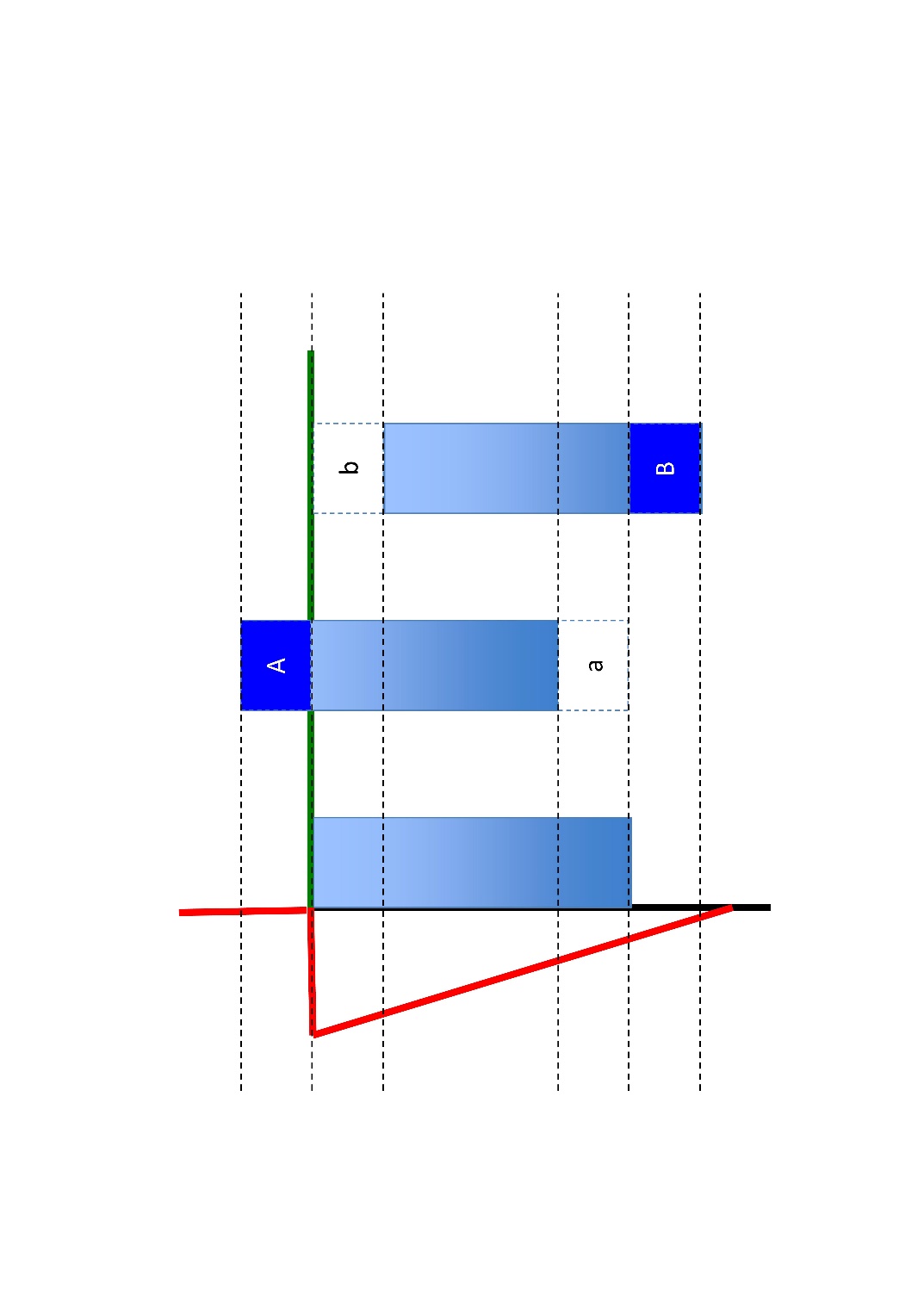


### Supplementary Figure 2. A proof of the optimal aim point in a uniform distribution.

Three blue rectangles represent uniform distributions which span the same range but are set at different locations. We assume that the reward region defined by the red lines is longer than the support of the uniform distribution as it is in the experiments. The upper limit of the left uniform distribution is on the green boundary line. We will prove that this is the location that maximizes expected gain. If we shift the left distribution upwards as shown in the middle figure, we effectively replace the region “a” (rewarded) by the region “A” (not rewarded) and thereby we reduce expected gain. On the other hand, if we shift the left distribution downwards as in the rightmost figure, we replace the region “b” (larger rewards) by the region “B” (smaller rewards) and again we can only reduce expected gain. The location of the distribution in the leftmost figure is the location maximizing expected gain.


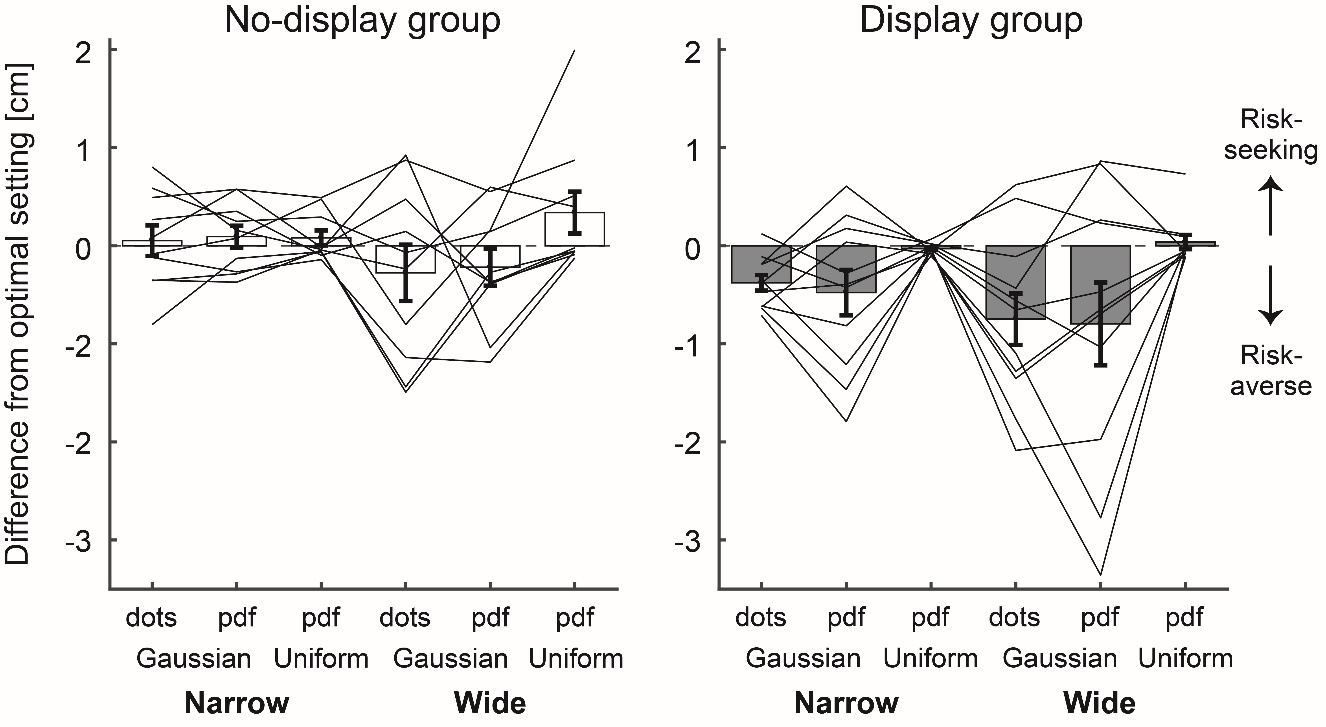


### Supplementary Figure 3. Singed difference of subjective and theoretical optimal aim points in the full information task.

The results of six distributions (three types of distribution ×two width of distribuion) are plotted for the no-display and display groups. A positive value indicates that the participants set the distribution closer to the penalty boundary than the theoretical optimal aim point, whereas a negative value indicates that they set the distribution further from the boundary than the optimal point. Thin lines represent the difference for each participant. Bars and error bars represent the mean difference and the standard errors of the mean, respectively. A three-way (2 [groups] $\times$ 3 [sample of Gaussian, PDF of Gaussian, and PDF of uniform distribution] $\times$ 2 [narrow and wide]) mixed-effects ANOVA showed a main effect of a type of distribution (*F* [2, 38] = 5.84, *p* = 0.006, $\eta^{2}$ = 0.08) and a significant interaction of a type of distribution and a width of distribution (*F* [2, 38] = 6.55, *p* = 0.004, $\eta^{2}$ = 0.03). In the wide distribution condition, the signed difference in the sampled dots of a Gaussian distribution was significantly lower than that in the PDF of a uniform distribution (*p* = 0.012, Bonferroni correction). Further, in the same condition, the signed difference in the PDF of a Gaussian distribution was significantly lower than that in the PDF of a uniform distribution (*p* = 0.017, Bonferroni correction). ANOVA did not yield a main effect of a group (*F* [1, 19] = 4.22, *p* = 0.0054, $\eta^{2}$ = 0.01), a main effect of the width (*F* [1, 19] = 2.93, *p* = 0.10, $\eta^{2}$ = 0.01), significant interaction of the group and the width (*F* [1, 19] = 0.17, *p* = 0.69, $\eta^{2}$ = 0.00), significant interaction of the group and the type (*F* [2, 38] = 0.76, *p* = 0.47, $\eta^{2}$ = 0.01), and significant interaction of the group, type, and width (*F* [2, 38] = 0.19, *p* = 0.83, $\eta^{2}$ = 0.00).


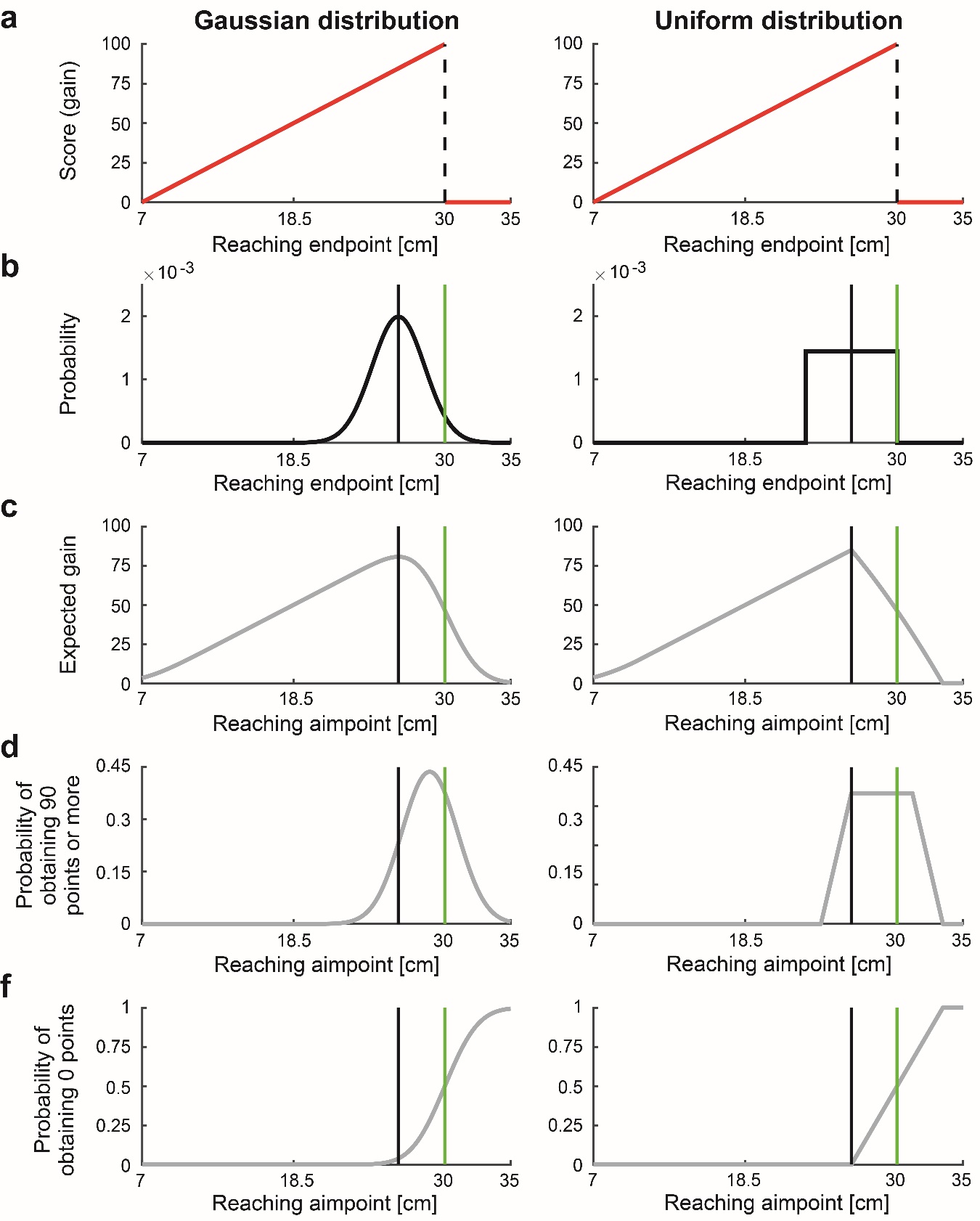


### Supplementary Figure 4. Simulation of expected gain in a Gaussian and uniform distribution.

**(a)** Asymmetric gain function. **(b)** Probability density function of a Gaussian and uniform distribution. **(c)** Expected gain as a function of the set (aim) point for distribution. See Model assumptions for the details of the calculation. **(d)** Probability of obtaining 90 points or more given the set point. **(f)** Probability of obtaining 0 points (mistrial) given the set point. Those probabilities were calculated by integrating the probability density function with the region from 90 to 100 points (27.7 to 30 cm) or the region after 100 points (after 30 cm), i.e., $P_{>90}\left( E \right)= \int_{27.7}^{30} G\left( e \right)･P(e|E)de$ and $P_{0}\left( E \right)= \int_{30}^{\infty} G\left( e \right)･P(e|E)de$. In panels from **(b)** to **(f)**, a green line denotes a penalty boundary and a vertical black line denotes the theoretical optimal set point which maximizes the expected gain given distribution in the full information task. In a Gaussian distribution, the probability of obtaining 90 points or more and that of obtaining 0 points continue to increase as the distribution is set to be closer and closer to the penalty boundary. Therefore, there exists a trade-off between a chance of higher reward and risk of penalty. On the other hand, in a uniform distribution, the probability of obtaining 90 points or more first increases and does not increase after a right edge of the uniform distribution is set just on the penalty boundary. After this point, the probability of obtaining 0 points starts to increase. Therefore, there does not exist the trade-off shown in a Gaussian distribution. The optimal strategy is more obvious because it requires one to adjust the right edge of the uniform distribution to the boundary line.
